# A Direction-Aware Dual-Branch Network for Surface-Strand Orientation Segmentation of Oriented Strand Board

**DOI:** 10.3390/s26165055

**Published:** 2026-08-09

**Authors:** Changyu Zhang, Yanyi Liu, Yin Wu

**Affiliations:** College of Information Science and Technology & Artificial Intelligence, Nanjing Forestry University, Nanjing 210037, China

**Keywords:** oriented strand board, semantic segmentation, dual-branch network, direction-vector auxiliary supervision, angle-class segmentation, edge deployment

## Abstract

The angular distribution of surface-strands in oriented strand board (OSB) is closely associated with board mechanical properties and mat formation quality. By acquiring surface images through vision sensing and combining them with deep learning-based segmentation, the angle classes of OSB surface-strands can be segmented and statistically analyzed automatically. However, OSB surface images contain complex strand textures, blurred boundaries, local adhesion between adjacent strands, and subtle differences among neighboring angle classes. To address these challenges, this study proposes a direction-aware dual-branch semantic segmentation network (DiBiNet) for pixel-level segmentation of surface-strand angle classes. OSB surface images were collected using a Hikrobot MV-CE120-10UC color industrial camera, and an 11-class dataset was constructed, including the background and ten angle classes from 0° to 90°. The samples were cropped to 512 × 512 pixels, and an improved angle-semantic-consistent Copy–Paste strategy was used to augment the training data. DiBiNet enhances directional feature representation through a Directional Strip Detail Enhancement Module, improves semantic feature modeling by combining MobileNetV3-Small with a DS-MobileViT Block, and fuses the two branches through a Bilateral Gated Fusion Module. Considering the continuity among angle classes, Direction Vector Auxiliary Supervision is introduced to map discrete angle labels into continuous direction vectors, thereby improving discrimination among neighboring classes. Experiments on the self-constructed dataset show that DiBiNet achieves a mean Intersection over Union (mIoU) of 0.8532, an overall pixel accuracy (Acc) of 0.8823, and a Dice coefficient of 0.8623, outperforming several representative semantic segmentation models. After 8-bit integer (INT8) + 16-bit floating-point (FP16) mixed quantization, the model achieves a neural processing unit (NPU) inference speed of 34.0 frames per second (FPS) on the RK3588 platform, demonstrating its potential for vision-based sensing and edge AI inspection.

## 1. Introduction

Oriented strand board (OSB) is an important wood-based composite material with high material utilization, good dimensional stability, and favorable mechanical properties. It is widely used in construction, furniture, and decoration. The performance of OSB is related not only to raw materials, adhesive application, and hot-pressing processes, but also to the arrangement and orientation distribution of surface-strands [1,2,3,4]. The orientation and local deflection of surface-strands affect the board’s modulus of rupture, modulus of elasticity, and overall structural stability. Therefore, accurate recognition of the orientation state of OSB surface-strands is important for mat formation quality assessment and production-process optimization.

**Figure 1:**
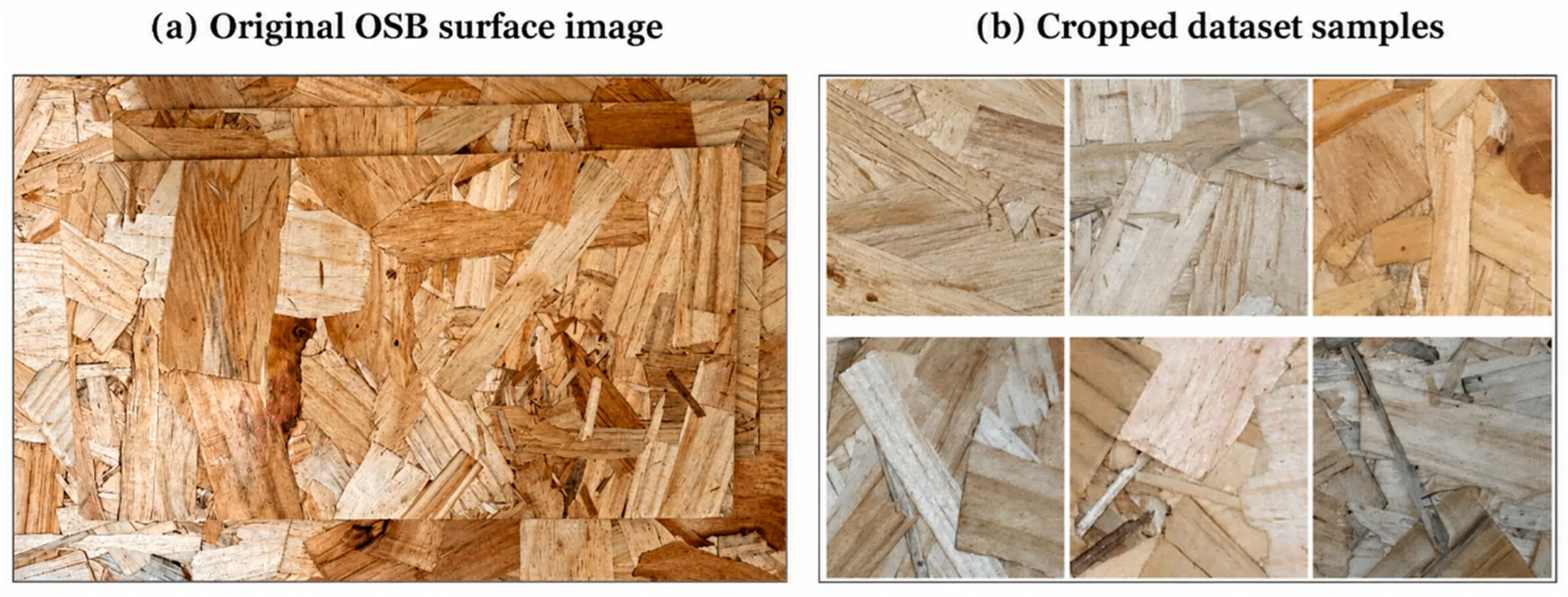
Original oriented strand board (OSB) surface image and local cropped samples.

In this study, a Hikrobot MV-CE120-10UC color industrial camera (Hangzhou Hikrobot Co., Ltd., Hangzhou, China) was used to acquire OSB surface images. These images contain the texture, boundary, and orientation information of surface-strands and were used for subsequent pixel-level segmentation and angle-distribution analysis. The original OSB surface image and local cropped samples are shown in Figure 1.

**Figure 2:**
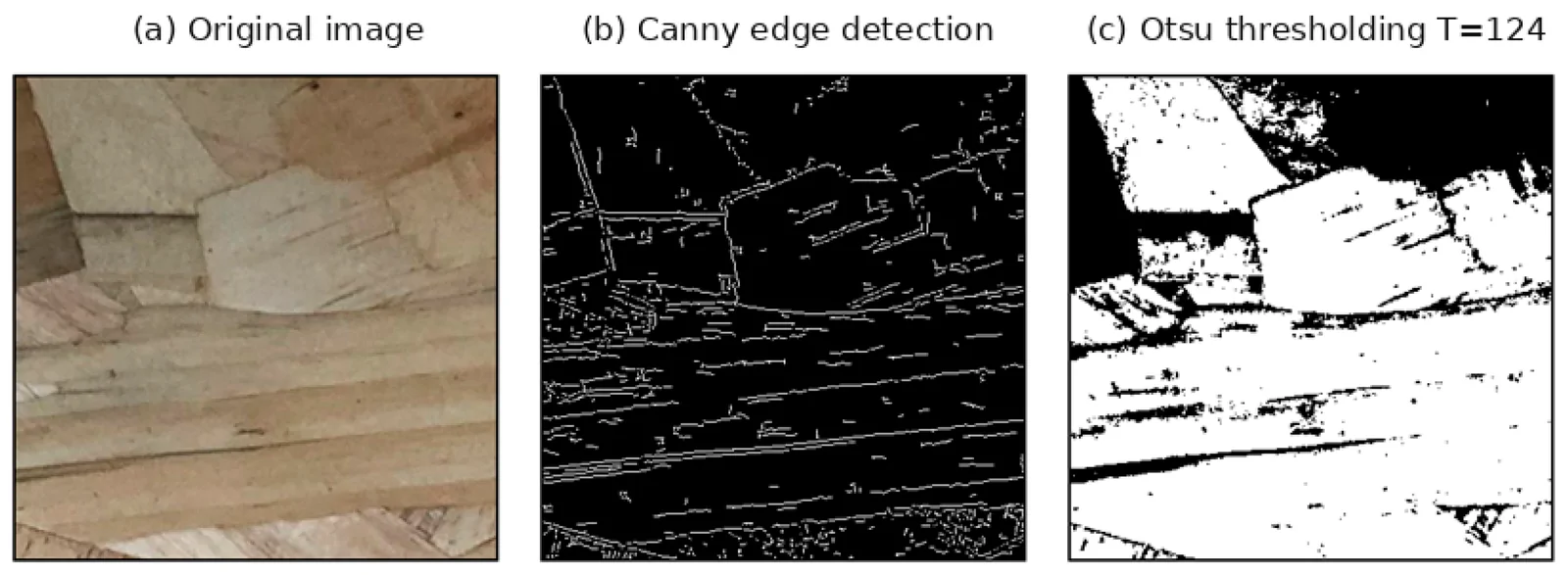
Comparison of traditional image segmentation methods.

As shown in Figure 1, the OSB surface contains numerous strands with different orientations, irregular shapes, and overlapping structures. The images also exhibit strong wood-grain texture interference and complex spatial patterns. Traditional OSB surface inspection methods mainly rely on manual observation, offline measurement, or image-processing methods based on edges, thresholds, and texture features. These methods can be effective when target shapes are regular and background interference is weak. However, when applied to OSB surface images with similar textures, blurred boundaries, occlusion, and local adhesion between adjacent strands, handcrafted features alone are often insufficient for robust strand extraction. The results of traditional image-segmentation methods are shown in Figure 2.

As shown in Figure 2, Canny edge detection [5] is easily affected by wood-grain texture interference, producing many false edges and boundary discontinuities. Otsu threshold segmentation [6] mainly separates regions according to grayscale differences and therefore struggles to distinguish strands with similar textures or overlapping structures. As a result, traditional image-processing methods cannot meet the requirements of pixel-level segmentation of OSB surface-strand angle classes. In contrast, deep learning-based semantic segmentation models can automatically learn multi-level image features from data and perform pixel-level classification, making them more suitable for OSB surface-strand recognition under complex textured backgrounds [7].

OSB surface-strand angle segmentation differs from ordinary semantic segmentation because its class labels indicate not only the pixel region but also the orientation angle of the strand. On the one hand, strands are mostly slender flakes, and boundary continuity and directional texture directly affect segmentation quality. On the other hand, angle classes such as 0°, 10°, and 20° are not completely independent, but instead exhibit a continuous relationship. If only a conventional semantic segmentation network and cross-entropy loss are used, the model tends to treat the angle classes as independent discrete labels and fails to exploit the relative distance among them.

Based on these characteristics, this study proposes a direction-aware dual-branch semantic segmentation network (DiBiNet). Inspired by dual-branch segmentation architectures [8,9], DiBiNet preserves strand boundaries and texture information through a high-resolution detail branch, extracts high-level semantic features through a low-resolution semantic branch, and incorporates angular-continuity constraints to improve segmentation stability for OSB surface-strand angle classes. The main contributions of this study are as follows:

(1) An OSB surface-strand angle semantic segmentation dataset is constructed. The dataset contains the background class and ten angle classes from 0° to 90°. Image cropping and angle-semantic-consistent Copy–Paste are used to augment the training samples, providing a data foundation for OSB surface-strand angle segmentation.
(2) DiBiNet, a direction-aware dual-branch semantic segmentation network, is proposed. The model enhances local directional details through the Directional Strip Detail Enhancement Module (DSDM) and supplements global semantic information through the DS-MobileViT Block. A bilateral gated fusion structure is used to integrate the detail and semantic features, thereby improving segmentation performance in complex textured scenes.
(3) A direction-vector auxiliary supervision method is introduced. It maps discrete angle labels into a continuous direction-vector space, enabling the model to learn the continuity among adjacent angle classes and improving the stability of angle-class segmentation. Experimental results show that the proposed method outperforms several representative semantic segmentation models in terms of mean Intersection over Union (mIoU), overall pixel accuracy (Acc), and Dice.

## 2. Related Work

In recent years, vision sensing and deep learning have been increasingly applied to wood and OSB inspection. Hong et al. [10] combined the Segment Anything Model, YOLOv5, and a minimum-area bounding-rectangle algorithm to track the orientation and dimensions of strands on an OSB production line, enabling continuous measurement of strand instances. However, this method requires relatively complete and distinguishable strand instances and is not directly applicable to pixel-level angle-class segmentation in severely overlapping surface regions. Zhu et al. [11] used image and depth information for wood-surface damage-defect segmentation, reducing interference from stains and mineral streaks; however, their method requires additional depth acquisition and mainly separates defects from the background. Mou et al. [12] developed a lightweight improvement of DeepLabv3+ for wood-based panel image segmentation, achieving a favorable balance between model accuracy and computational cost, but without further considering the continuity among angle classes.

Directional information has also been used in semantic-segmentation feature modeling. DWin-HRFormer [13] employs directional-window self-attention to extract direction-sensitive structural features while controlling attention computation. This method mainly strengthens directional responses at the feature-extraction level, whereas the 0–90° classes in this study also have an explicit angular order and neighborhood relationship. Therefore, in addition to enhancing directional texture features, DiBiNet constrains the relationship among adjacent angle classes through direction-vector auxiliary supervision.

In the field of image restoration, Sultan et al. [14] proposed DSCH-Net, which combines diffusion priors, state-space modeling, and contextual feature interaction for direction-aware dehazing of remote-sensing images. Their study indicates that directional and contextual information can improve feature-representation stability under spatially nonuniform degradation. Because DSCH-Net is designed for image restoration and its output objective differs from the pixel-level segmentation of strand angle classes considered here, it is cited as related work on directional-information modeling.

**Figure 3:**
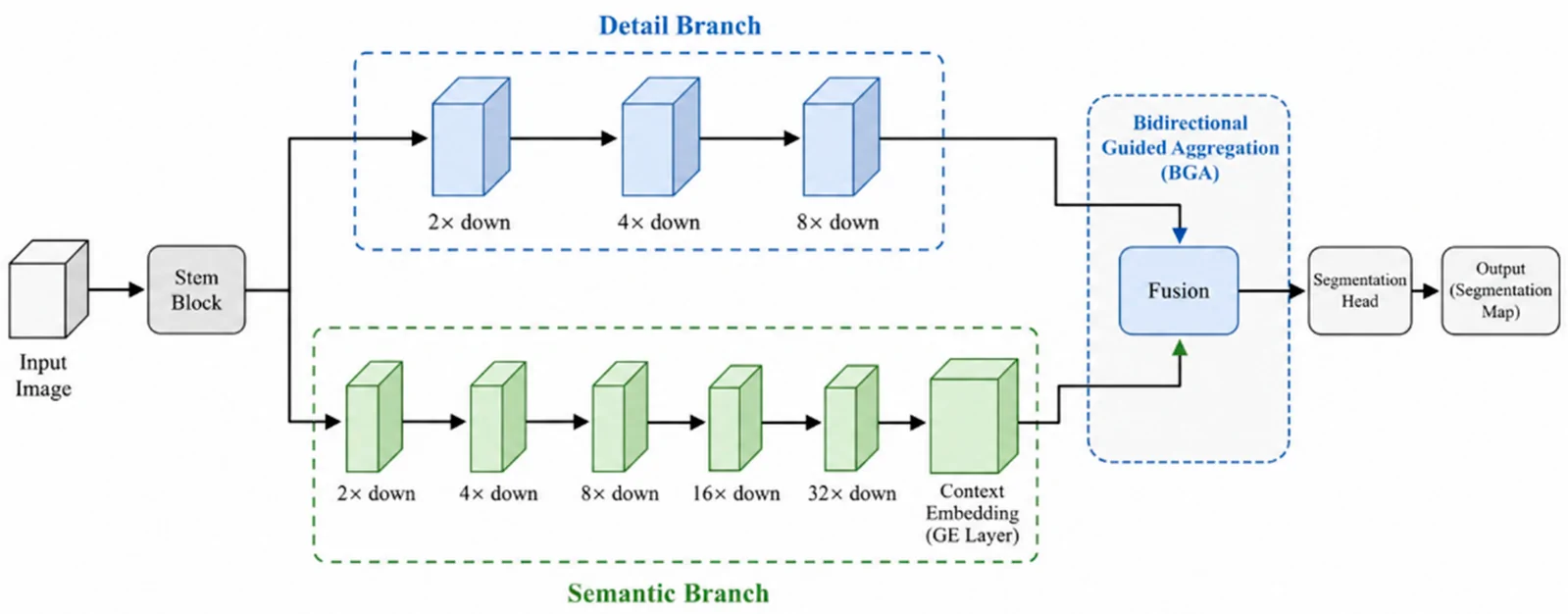
Structure of Bilateral Segmentation Network V2 (BiSeNetV2).

Semantic segmentation is an important task in computer vision. Its goal is to assign a class label to each pixel in an image, thereby obtaining segmentation results with both spatial and semantic information. In tasks involving complex textures and fine-grained class definitions, blurred boundaries, similar local structures, and small inter-class differences increase segmentation difficulty. The model must therefore balance feature-representation capacity and computational efficiency. Among real-time semantic segmentation networks, the Bilateral Segmentation Network (BiSeNet) series uses a dual-branch structure to jointly preserve spatial details and semantic information [8,9]. The detail branch retains high-resolution boundary and texture features, while the semantic branch expands the receptive field through downsampling and extracts contextual information before feature fusion. This structure achieves a good balance between segmentation accuracy and inference efficiency. However, in scenes with complex textures and low class separability, relying only on convolutional structures may still lead to insufficient global-dependency modeling. The structure of BiSeNetV2 is shown in Figure 3.

To enhance global contextual representation, Transformer architectures have been introduced into vision tasks [15,16]. Their self-attention mechanism can model long-range dependencies among different spatial positions and compensate for the limited local receptive field of convolution. However, standard Transformers incur high computational costs and are not well suited to lightweight deployment. MobileViT combines the local feature-extraction ability of convolution with the global modeling capability of Transformers, enhancing contextual representation while maintaining relatively low computational complexity [17]. In addition, MobileNetV3-Small has a lightweight design and is commonly used for feature extraction in mobile and edge vision tasks [18]. Therefore, combining a dual-branch structure, a lightweight backbone, and local-global modeling is beneficial for simultaneously preserving boundary details, improving semantic representation, and maintaining edge-deployment efficiency in OSB surface-strand angle segmentation.

## 3. Dataset and Methods

### 3.1. OSB Surface Image Acquisition and Angle-Class Definition

Surface images of hot-pressed oriented strand board were acquired using a color industrial camera. During image acquisition, the board surface was photographed from a top-down view, with the camera optical axis approximately perpendicular to the board surface. This setup reduces perspective distortion and enables strand orientations to be consistently defined relative to the horizontal direction of the image.

The OSB surface contains strands with different orientations, irregular shapes, and overlapping structures, together with strong wood-grain interference and complex spatial patterns. Therefore, pixel-level annotation of strand regions and their orientation classes is required. The acquired images were subsequently cropped into 512 × 512 sub-images for pixel-level annotation and dataset construction.

**Figure 4:**
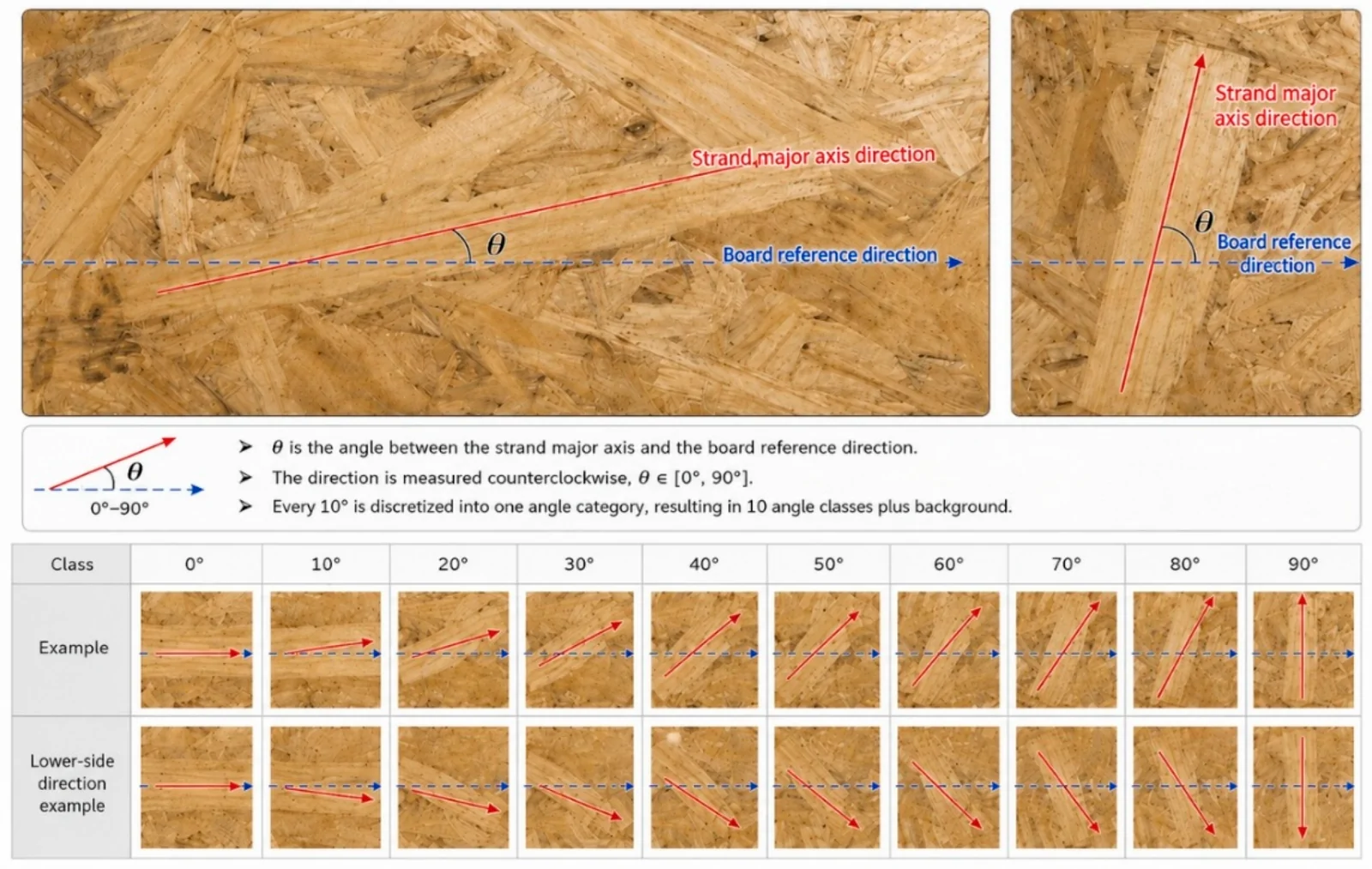
Schematic diagram of surface-strand orientation categories.

According to the inclination angle of each strand relative to the board reference direction, surface-strand orientations are divided into ten angle classes: 0°, 10°, 20°, 30°, 40°, 50°, 60°, 70°, 80°, and 90°. A background class is additionally defined, resulting in 11 semantic classes in total. The angle-class definition is shown in Figure 4.

Because the original images have high resolution, directly using them for network training would cause excessive GPU memory consumption and make it difficult to standardize the model input size. Therefore, the original images are cropped into 512 × 512 pixel sub-images for subsequent training, validation, and testing. During annotation, labelme v4.6.0 [19] is used to outline each strand region with polygons, and the corresponding class label is assigned according to the principal orientation of the strand. After annotation, the files are converted into class-index label maps, where the background class corresponds to pixel value 0 and the angle classes from 0° to 90° correspond sequentially to pixel values 1–10.

The principal orientation of each annotated strand is determined from its dominant longitudinal axis. Specifically, the annotator identifies the longest visible extension direction according to the elongated contour of the strand polygon and its internal wood-grain texture. The orientation angle θ is defined as the acute angle between this longitudinal axis and the horizontal image axis; therefore, axes differing by 180° are treated as equivalent. The estimated angle is then assigned to the nearest 10° class within the range of 0–90°. For partially occluded or irregularly shaped strands, the longest visible longitudinal extension within the annotated region is used as the reference.

The dataset contains 6770 annotated 512 × 512 sub-images and is divided into training, validation, and test sets at a ratio of 7:2:1. Each Labelme polygon is counted as one annotated strand instance, and the number and proportion of annotated instances in each angle class are summarized in Table 1. These cropped samples originate from approximately 200 original OSB surface images with a resolution of 3024 × 4032. To prevent similar textures from the same source image from affecting model evaluation, the dataset is split at the original-image level. All sub-images cropped from the same original image are assigned exclusively to the training, validation, or test set and are not shared across subsets.

To further analyze the class distribution of strands with different orientations, each Labelme polygon carrying an angle label is counted as one strand instance. Because the background class does not correspond to an independent strand instance, it is excluded from the instance-count and proportion statistics. The numbers and proportions of annotated instances for the angle classes from 0° to 90° are listed in Table 1.

As shown in Table 1, the strand-instance distribution differs among the angle classes. The 0–20° classes account for relatively high proportions, whereas the 70–90° classes are less frequent. Nevertheless, every angle class contains a substantial number of annotated instances, providing a basis for model training and performance evaluation across different strand orientations.

### 3.2. Angle-Semantic-Consistent Copy–Paste Data Augmentation

Based on conventional Copy–Paste augmentation [20], this study adopts an angle-semantic-consistent Copy–Paste strategy to augment the training samples. In the OSB surface-strand angle segmentation dataset, class labels not only represent spatial regions but also correspond to the included angle between the strand principal axis and the horizontal direction. Therefore, when a strand region is rotated, its angle class should also change accordingly. Based on this property, geometric rotation can generate new angle-class samples as long as the labels are synchronously updated, making it well suited to the dataset definition used in this study.

Specifically, a relatively complete connected strand region is first extracted from the source label. The region is then rotated by an integer multiple of 10°, and the acute angle between its principal axis and the horizontal direction is recalculated to obtain the updated angle class. Finally, the rotated region is pasted into a target image, and the corresponding semantic label is synchronously updated.

Let the angle corresponding to the original angle class be θ and the rotation angle be Δθ. The augmented angle and class index can be expressed as follows:

(1) $$\theta^{\prime }=\theta +\Delta \theta$$

(2) $$c^{\prime }=\theta^{\prime }/10+1$$

where *c*′ denotes the updated class index. Since the background class corresponds to pixel value 0, the angle classes from 0° to 90° correspond sequentially to pixel values 1–10. When *θ′* exceeds the range of 0–90°, it is folded into the acute angle relative to the horizontal direction according to the undirected-axis relationship.

The synthesis process of the augmented image and augmented label can be expressed as follows:

(3) $$I_{aug}=M⊙I_{p}+\left(1-M\right)⊙I_{t}$$

(4) $$Y_{aug}=M⊙Y_{p}+\left(1-M\right)⊙Y_{t}$$

where *I_t_* and *Y_t_* denote the target image and its label, respectively; *I_p_* and *Y_p_* denote the pasted region image and label after rotation and category updating, respectively; *M* denotes the mask of the pasted region; and ⊙ denotes element-wise multiplication.

**Figure 5:**
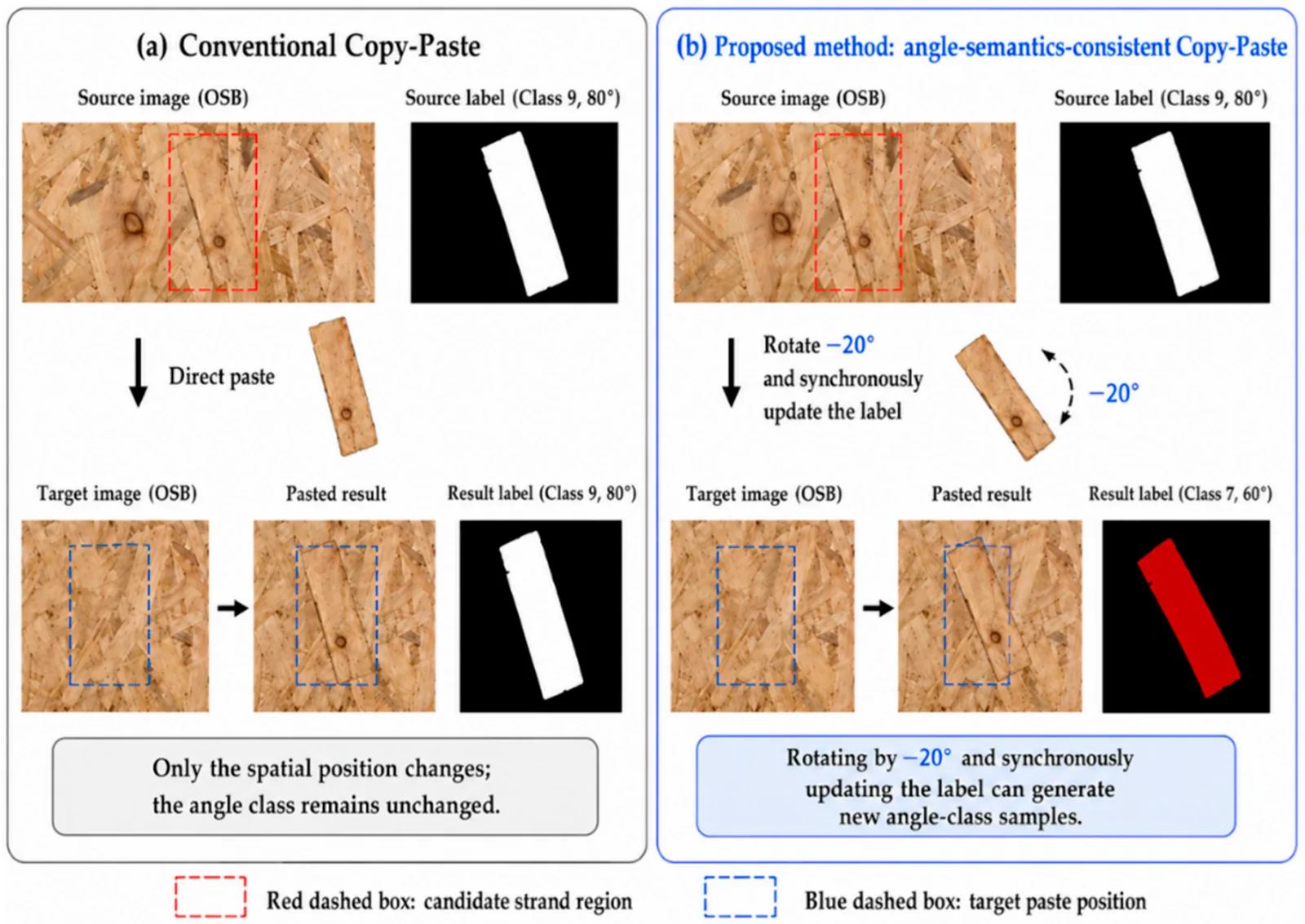
Comparison between conventional Copy–Paste and angle-semantic-consistent Copy–Paste.

Compared with conventional Copy–Paste, angle-semantic-consistent Copy–Paste can generate new angle-class samples without additional manual annotation while maintaining consistency between the augmented images and labels. This method exploits the local recombinability of OSB surface-strand regions, the clearly defined discrete angle classes, and the similar background textures among samples, helping to improve the model’s ability to recognize strands with different orientations. The workflow is shown in Figure 5.

After Copy–Paste, additional overlap or boundary discontinuities may occur in local regions. Because strand overlap is inherent to OSB surfaces and the source and target images have similar textures, the synthesized samples retain a certain degree of similarity to real surfaces. Nevertheless, some pasted regions may still exhibit unnatural arrangements. To reduce this effect, relatively complete connected strand regions are selected, and both the pasted region and its corresponding label are updated synchronously.

### 3.3. Overall Structure of DiBiNet

**Figure 6:**
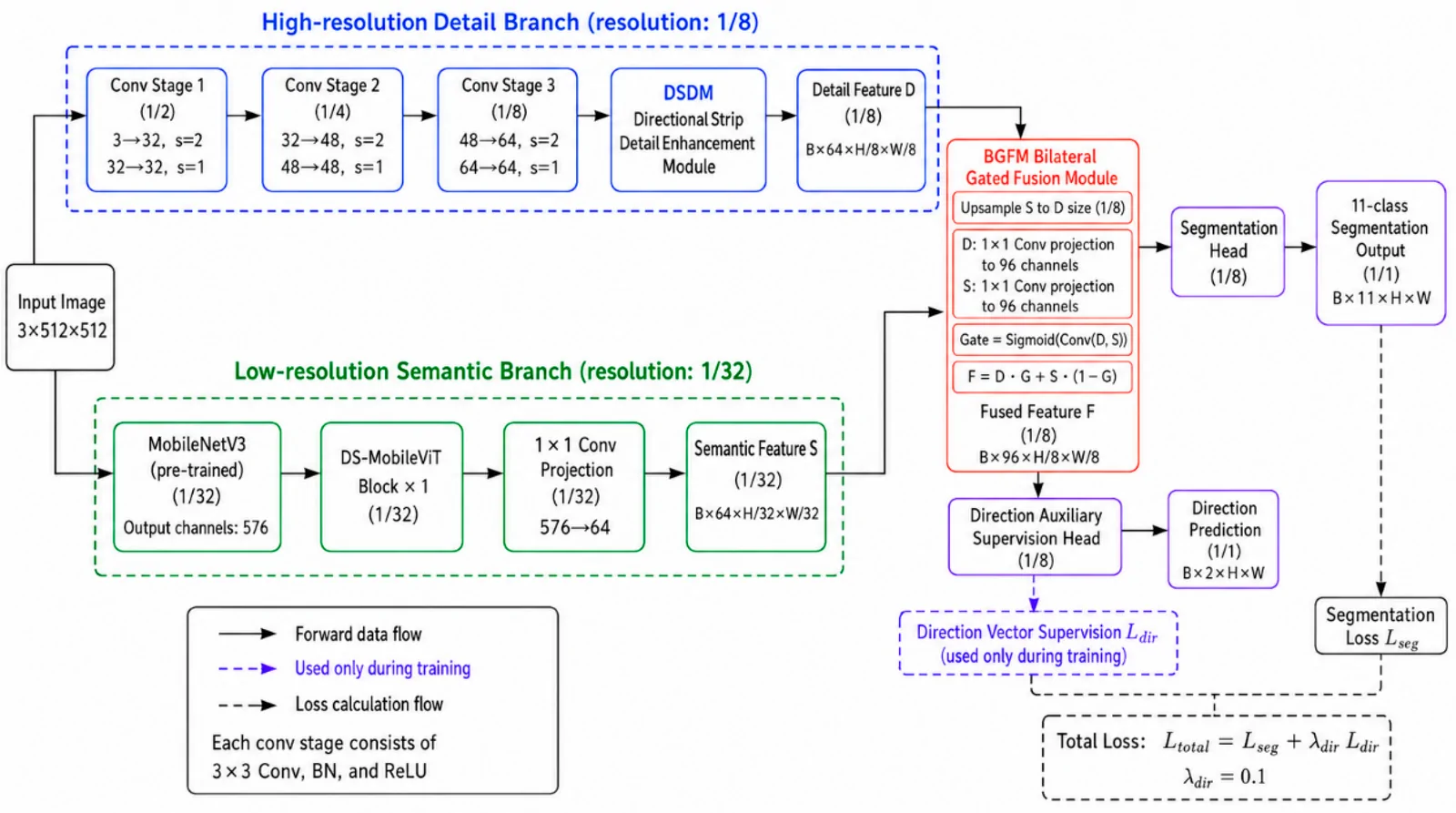
Architecture of the proposed direction-aware dual-branch semantic segmentation network (DiBiNet).

The overall structure of the proposed DiBiNet is shown in Figure 6. The model adopts a dual-branch semantic segmentation architecture consisting of a high-resolution detail branch, a low-resolution semantic branch, the Bilateral Gated Fusion Module (BGFM), a segmentation prediction head, and a direction-vector auxiliary supervision branch used only during training. The input image size is 3 × 512 × 512, and the model outputs an 11-class semantic segmentation map, including the background class and ten strand angle classes from 0° to 90°.

In OSB surface images, strand regions usually exhibit slender shapes, irregular forms, overlap, and blurred boundaries. If only low-resolution semantic features are used, boundary details are easily lost; if only high-resolution shallow features are used, it is difficult to stably distinguish angle classes with similar textures. Therefore, DiBiNet extracts complementary information through the detail branch and the semantic branch. The detail branch mainly preserves boundary, texture, and spatial-position information, and introduces the Directional Strip Detail Enhancement Module (DSDM) at the end to enhance slender strand boundaries and directional texture representation. The semantic branch uses MobileNetV3-Small as the backbone and introduces a DS-MobileViT Block at the low-resolution feature layer to supplement global contextual information and improve the discrimination of similar angle classes and long-range strand structures.

During feature fusion, BGFM adjusts the detail-branch and semantic-branch features to the same scale and performs gated fusion, enabling the model to adaptively assign weights to detail and semantic information according to regional feature responses. The fused feature is fed into the segmentation prediction head to generate 11-class segmentation results, which are then upsampled to the input image size. In addition, during training, a direction prediction head is derived from the fused feature for direction-vector auxiliary supervision. This branch participates only in training and is not used during inference or deployment, so it does not increase the actual inference cost.

### 3.4. Directional Strip Detail Enhancement Module

**Figure 7:**
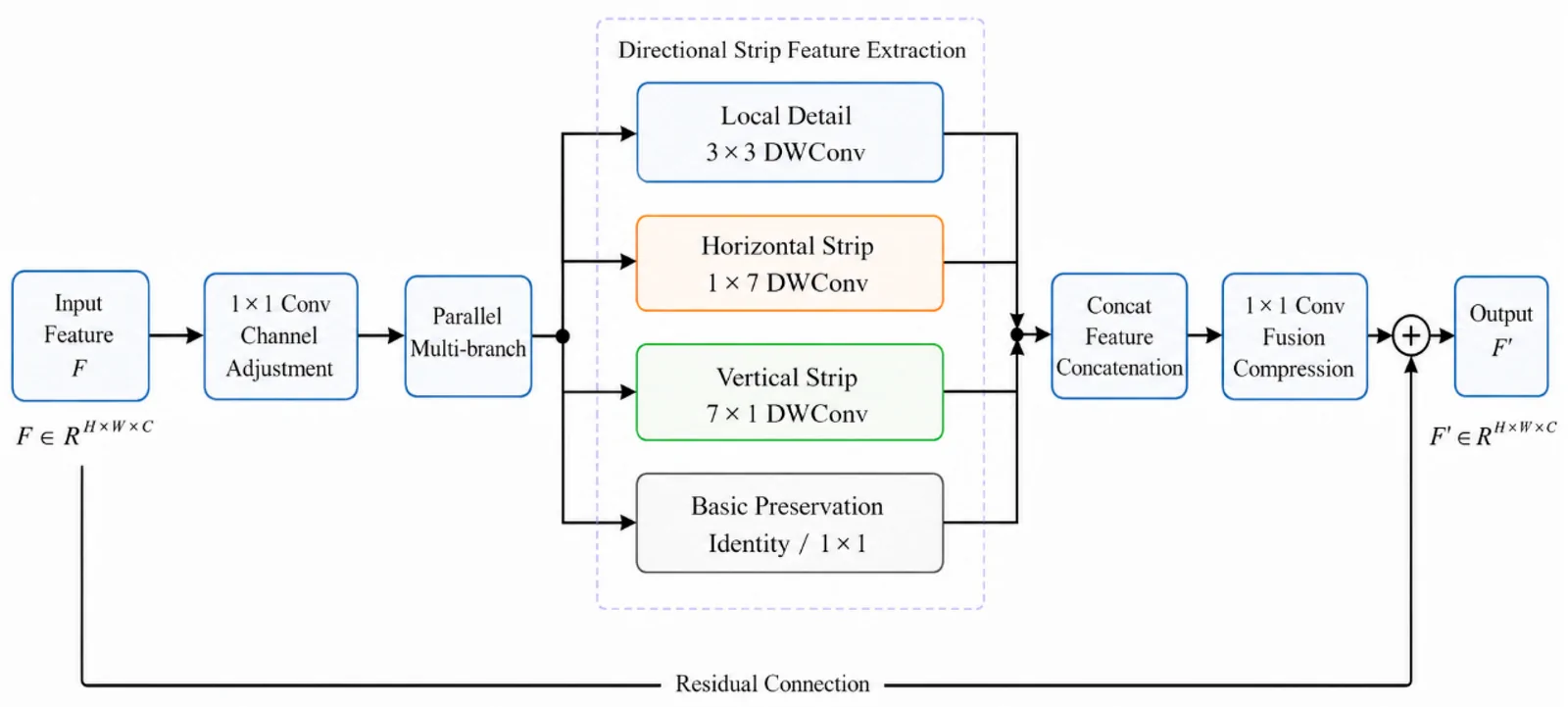
Structure of the Directional Strip Detail Enhancement Module (DSDM).

OSB surface-strands are usually distributed as slender strip-like regions, and some strands show clear directional extension in space. Although ordinary 3 × 3 convolution can extract local edge and texture information, its receptive field is limited and its ability to represent elongated structures and directionally continuous boundaries is insufficient. To enhance the model’s perception of strand directional textures and slender boundaries, this study designs the Directional Strip Detail Enhancement Module (DSDM) at the end of the high-resolution detail branch. Its structure is shown in Figure 7.

DSDM consists of a local detail branch, a horizontal strip branch, a vertical strip branch, and a basic preservation branch. The local detail branch uses 3 × 3 depthwise convolution to extract local edge and texture features; the horizontal strip branch uses 1 × 7 depthwise convolution to enhance horizontally continuous structures; the vertical strip branch uses 7 × 1 depthwise convolution to enhance vertically continuous structures; and the basic preservation branch retains the original input feature. The multi-branch features are concatenated along the channel dimension, fused and compressed through 1 × 1 convolution, and then connected with the input feature through a residual connection. This design enhances directional details while preserving the basic texture information.

This process can be simplified as:

(5) $$F_{out}=Conv_{1\times 1}\left(\left[F_{3\times 3},F_{1\times 7},F_{7\times 1},F_{id}\right]\right)+F_{in}$$

where F_3×3_, F_1×7_, F_7×1_, and F_id_ denote the outputs of the local detail branch, horizontal strip branch, vertical strip branch, and basic preservation branch, respectively; [·] denotes concatenation along the channel dimension; and Fin denotes the input feature.

With this structure, DSDM enhances the detail branch’s ability to represent local directional structures without further reducing spatial resolution, thereby helping to alleviate blurred strand boundaries, local discontinuities, and incomplete segmentation of slender regions.

### 3.5. Low-Resolution Semantic Branch and DS-MobileViT Block

In OSB surface images, the same strand may span a large spatial range, and texture differences between adjacent angle classes are often subtle. Relying only on local convolutional features may therefore lead to unstable class prediction. To enhance the use of global contextual information, this study introduces the DS-MobileViT Block into the low-resolution semantic branch.

The semantic branch first uses MobileNetV3-Small as the backbone to progressively downsample the input image and extract semantic features. MobileNetV3-Small has a lightweight design and is suitable for subsequent edge deployment. A DS-MobileViT Block is then inserted after the high-level semantic feature at the 1/32 scale. By combining the local modeling capability of convolution with the global dependency modeling capability of Transformers, this module can establish relationships among different regions on a low-resolution feature map with relatively low computational cost.

**Figure 8:**
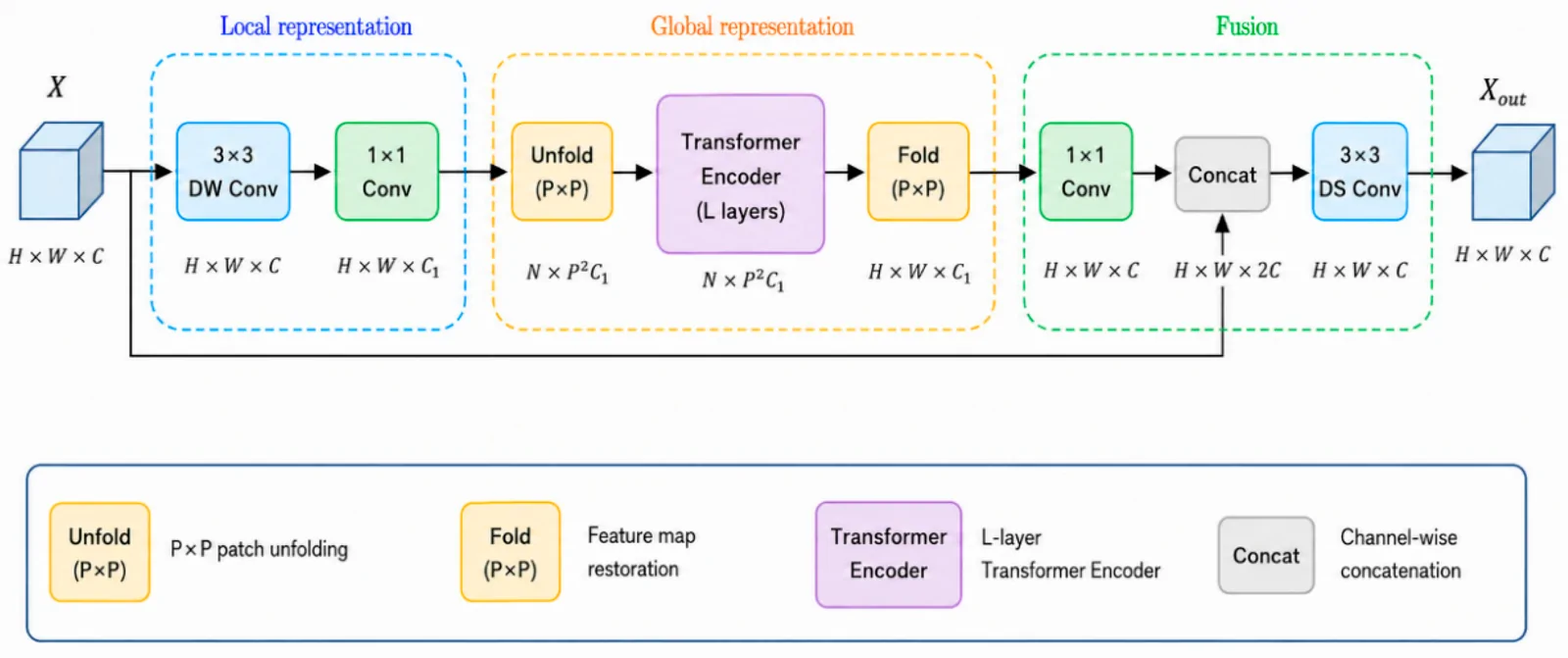
Structure of the DS-MobileViT Block.

The structure of the DS-MobileViT Block is shown in Figure 8. The module first uses depthwise separable convolution to extract local features and then reorganizes the two-dimensional spatial features into a sequence representation through Patch/Token unfolding for Transformer processing. The Transformer encoder is then used for global context modeling. Finally, the sequence features are folded back into a spatial feature map and fused with the local features.

### 3.6. Bilateral Gated Fusion Module

The high-resolution detail branch and the low-resolution semantic branch focus on different levels of feature information. The former retains rich boundary, texture, and spatial-position information but has relatively limited semantic discriminative ability; the latter has stronger class-semantic and contextual representation ability, but some boundary details may be lost because of its lower resolution. Therefore, the two types of features need to be effectively fused before segmentation prediction.

**Figure 9:**
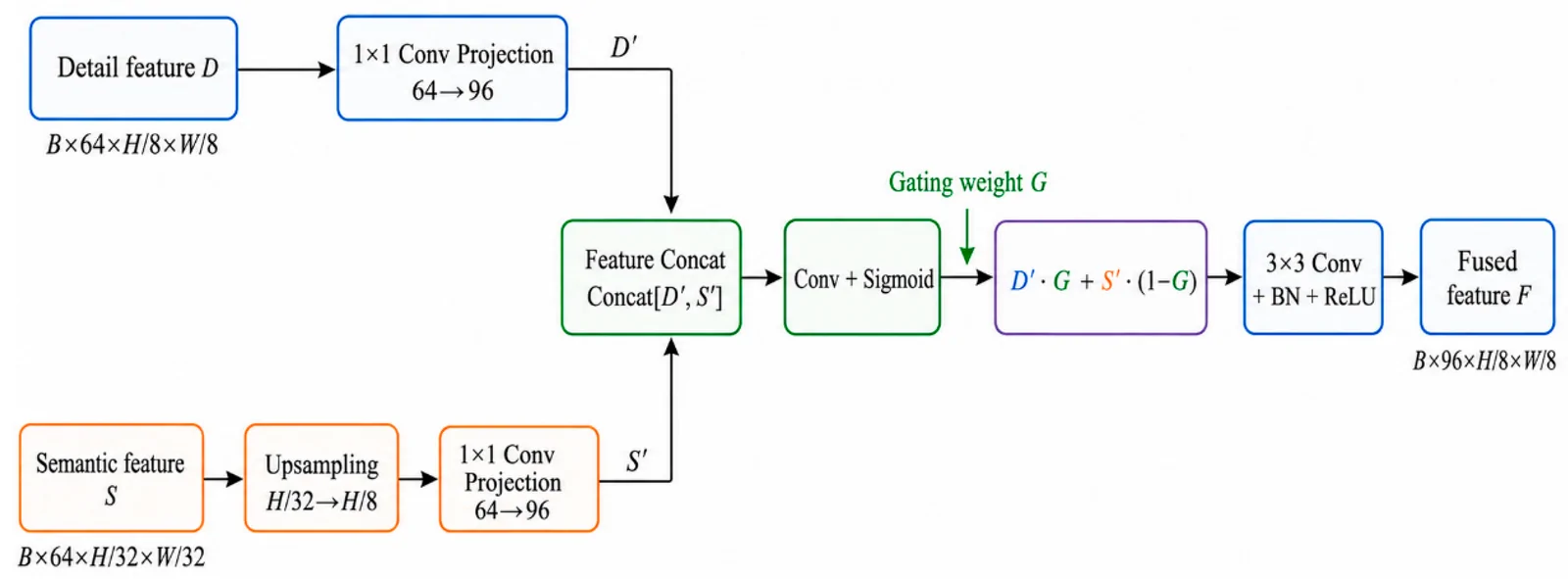
Structure of the Bilateral Gated Fusion Module (BGFM).

The Bilateral Gated Fusion Module (BGFM) is used to adaptively weight and fuse the detail and semantic features. Its structure is shown in Figure 9.

Let the mapped detail feature and semantic feature be Fd and Fs, respectively. The gate weight and fused feature can be expressed as:

(6) $$G=\sigma \left(f_{g}\left(\left[F_{d},F_{s}\right]\right)\right)$$

(7) $$F_{f}=G⊙F_{d}+\left(1-G\right)⊙F_{s}$$

where G denotes the gate weight, σ denotes the Sigmoid function, fg denotes the gate-generation convolution, [·] denotes concatenation along the channel dimension, and ⊙ denotes element-wise multiplication.

Compared with direct addition or simple concatenation, BGFM can introduce semantic contextual constraints while preserving detailed boundary information, thereby improving segmentation stability in complex junction regions and adjacent angle-class regions.

### 3.7. Direction Vector Auxiliary Supervision

**Figure 10:**
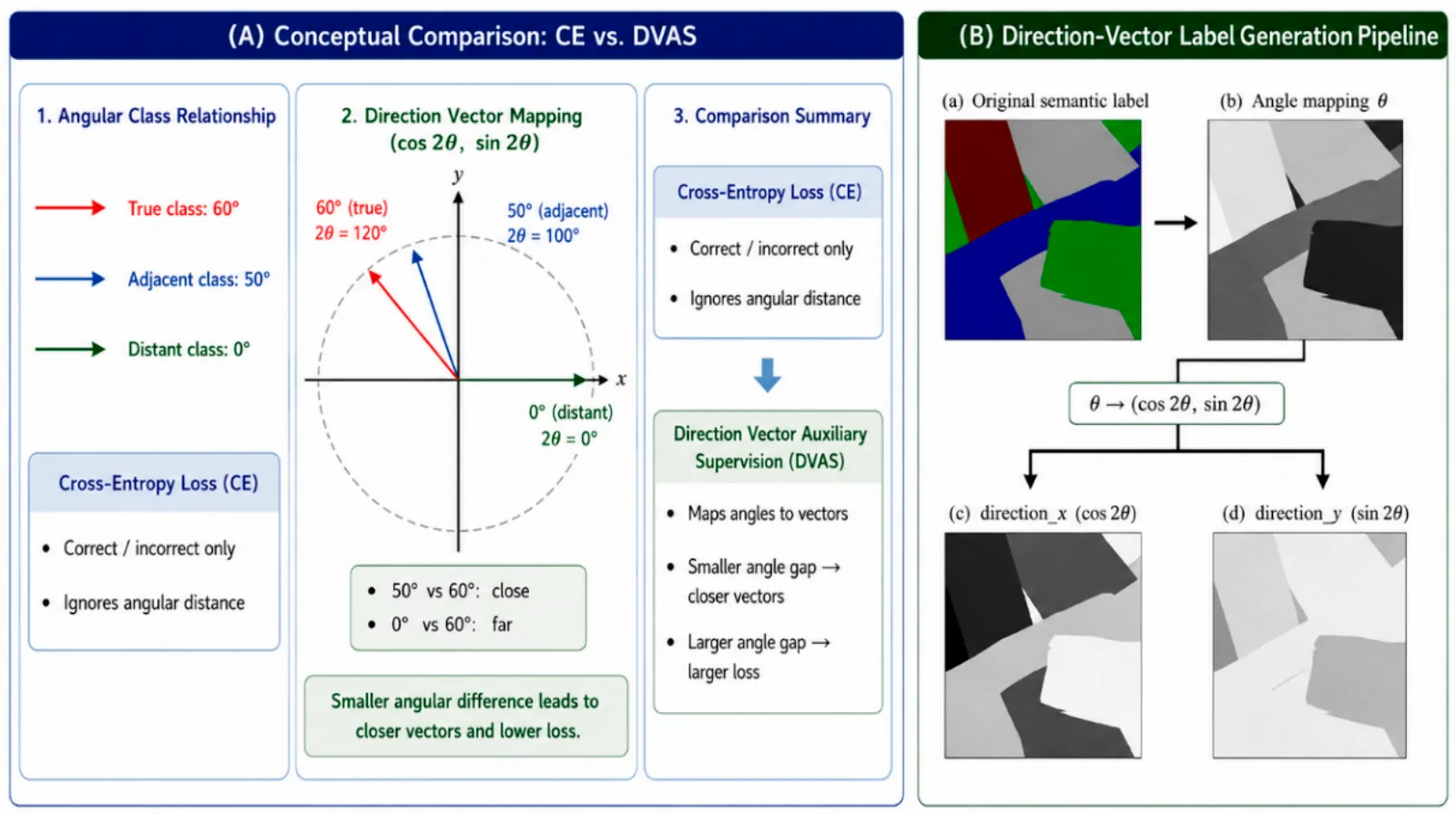
Comparison between cross-entropy supervision and Direction Vector Auxiliary Supervision (DVAS).

In ordinary semantic segmentation, different classes are usually treated as mutually independent discrete labels. However, in OSB surface-strand angle segmentation, categories such as 0°, 10°, 20°, …, and 90° have a clear angular-continuity relationship. For example, 50° is closer to 60° in orientation, whereas 0° and 90° are much farther apart. If only cross-entropy loss is used, the model cannot explicitly reflect this angular distance. Figure 10 illustrates the difference between ordinary cross-entropy supervision and direction-vector auxiliary supervision and presents the process of generating direction-vector labels.

To exploit this property, this study introduces Direction Vector Auxiliary Supervision (DVAS), which converts foreground angle labels into directional constraints so that the model can learn the relative relationships among angle classes in addition to pixel classification. Background regions do not have a clear orientation attribute and are therefore excluded from direction supervision. This constraint distinguishes minor angular deviations from obvious orientation errors, helping to reduce confusion among adjacent angle classes and improve prediction stability in complex boundary regions and texture-similar areas.

For foreground angle classes, the class label is first mapped to the corresponding angle θ, and then an undirected orientation representation is used to convert the angle into a two-dimensional direction vector:

t = (cos(2θ), sin(2θ))(8)

where *t* denotes the direction vector generated from the ground-truth label. Since strand orientation is better represented as an undirected axis rather than a directed arrow with a definite start and end point, using cos(2*θ*) and sin(2*θ*) avoids representation conflicts when 0° and 180° are equivalent. The background class has no explicit orientation attribute and is therefore excluded from the direction-loss calculation.

During training, the main segmentation head outputs 11-class semantic segmentation results, while the direction prediction head outputs two-dimensional direction vectors. The direction-vector auxiliary loss adopts a foreground-mask-based cosine distance:

(9) $$L_{dir}=\frac{1}{N_{f}}\sum_{i}\left[1-\cos\left(p_{i},t_{i}\right)\right]$$

where *N_f_* denotes the number of foreground pixels, *p_i_* denotes the predicted direction vector of the i-th foreground pixel, *t_i_* denotes the direction vector generated from the ground-truth label, and cos(*p_i_*, *t_i_*) denotes the cosine similarity between them. Since background pixels have no explicit orientation attribute, they are not included in the direction-loss calculation.

The final loss consists of the main segmentation loss and the direction vector auxiliary loss:

(10) $$L=L_{seg}+\lambda_{dir}L_{dir}$$

where *L*_seg_ consists of cross-entropy loss and Dice loss, *L*_dir_ denotes the direction-vector auxiliary loss, and *λ*_dir_ denotes the direction-loss weight. This auxiliary branch is used only during training to constrain feature learning. During inference, only the semantic segmentation output is retained; therefore, the auxiliary branch does not increase deployment inference cost.

## 4. Experimental Results and Analysis

### 4.1. Experimental Settings and Evaluation Metrics

All experiments are conducted under the same dataset split and training parameters to ensure a fair comparison among different models. The input image size is uniformly set to 512 × 512, and the dataset is divided into training, validation, and test sets at a ratio of 7:2:1. The model is trained using the Adam optimizer [21] with an initial learning rate of 1 × 10^−4^, a batch size of 8, and 200 training epochs. The main segmentation loss consists of cross-entropy loss and Dice loss [22]. The direction-vector auxiliary loss weight *λ*_dir_ is set to 0.1. This value is selected through a sensitivity comparison to balance the directional constraint and the primary semantic-segmentation objective: an excessively small weight provides insufficient directional guidance, whereas an excessively large weight may interfere with optimization of the main segmentation loss. The basis for this choice is presented in the weight-sensitivity experiment in Section 4.2.3. The experimental environment is listed in Table 2.

To comprehensively evaluate segmentation performance, Intersection over Union (IoU), mean Intersection over Union (mIoU), overall pixel accuracy (Acc), and the Dice coefficient are adopted. TP_c, FP_c, and FN_c denote the numbers of true-positive, false-positive, and false-negative pixels for class c, respectively. Their mathematical definitions are as follows:

(11) $${\mathrm{IoU}}_{c}=\frac{TP_{c}}{TP_{c}+FP_{c}+FN_{c}}$$

(12) $$\mathrm{mIoU}=\frac{1}{11}\sum_{c=0}^{10}IoU_{c}$$

(13) $$\mathrm{Acc}=\frac{\sum_{c=0}^{10}TP_{c}}{\sum_{c=0}^{10}\left(TP_{c}+FN_{c}\right)}$$

(14) $${\mathrm{Dice}}_{c}=\frac{2TP_{c}}{2TP_{c}+FP_{c}+FN_{c}}$$

Here, c denotes the class index and ranges from 0 to 10, corresponding to the background class and ten strand-angle classes. The mIoU evaluates the average overlap between the predicted and ground-truth regions across different classes, Acc reflects the overall classification accuracy over all pixels, and the Dice coefficient provides a complementary measure of regional similarity. The same evaluation procedure was consistently applied to all ablation and comparison experiments.

### 4.2. Ablation Experiments

#### 4.2.1. Ablation Experiment on Data Augmentation Strategies

To verify the effectiveness of the angle-semantic-consistent Copy–Paste data augmentation strategy, different data augmentation methods are compared under the same model structure and training parameters. The results are shown in Table 3. Conventional augmentation refers to basic image augmentation only; conventional Copy–Paste directly copies and pastes strand regions without changing their angle-class attributes; and angle-semantic-consistent Copy–Paste synchronously updates the class label after region rotation.

As shown in Table 3, all three metrics of conventional Copy–Paste are higher than those obtained using only basic augmentation, indicating that increasing the combinations of strand regions is beneficial to model training. However, conventional Copy–Paste changes only the spatial position and retains the original angle label after pasting, so it cannot generate samples for new angle classes. Angle-semantic-consistent Copy–Paste rotates the strand region while synchronously updating its label, increasing mIoU from 0.8482 to 0.8532, with corresponding improvements in Acc and Dice. These results show that synchronously updating the angle label with image rotation is more consistent with the dataset definition used in this study.

#### 4.2.2. Ablation Experiment on Model Modules

To verify the effectiveness of the model structure and auxiliary-supervision design, a basic dual-branch network is used as the baseline, and DSDM, the DS-MobileViT Block, and DVAS are progressively added for ablation experiments. The results of the incremental modules are shown in Table 4. Because BGFM is a core feature-fusion component, Section 4.2.4 further compares it independently with element-wise addition, channel concatenation followed by a 1 × 1 convolution, and attention-based fusion. All models use the same dataset split, training parameters, and angle-semantic-consistent Copy–Paste augmentation strategy, ensuring that the reported differences reflect the effects of the corresponding model structures or supervision methods.

Table 4 presents the results obtained after progressively adding each module. The basic dual-branch network achieves an mIoU of 0.8213. After DSDM is added, mIoU increases to 0.8312, with improved segmentation of slender regions and local boundaries. When the DS-MobileViT Block is added independently, mIoU reaches 0.8294, indicating that low-resolution global information also provides a performance gain. Using both modules increases mIoU further to 0.8483, showing that local directional features and global semantic features are complementary. Adding DVAS on this basis yields an mIoU of 0.8532 and an Acc of 0.8823, which are the best results in the table.

**Figure 11:**
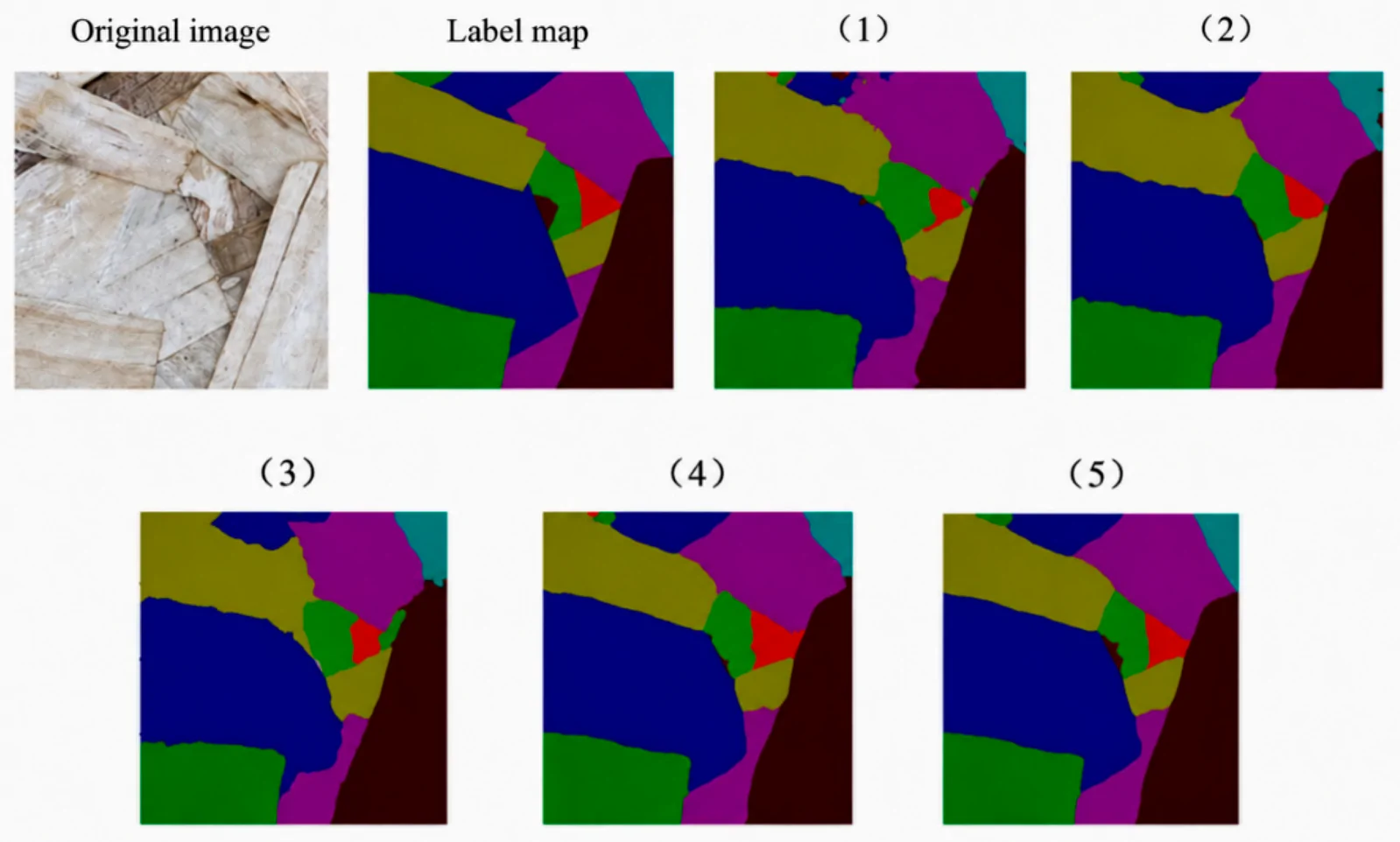
Visualization results of ablation experiments. Numbers (1)–(5) correspond to configurations (1)–(5) in Table 4; different colors represent different angle classes and follow the same color coding as the label map.

To further visually demonstrate the influence of different modules on the segmentation results, representative test samples are selected for visual comparison, as shown in Figure 11.

Figure 11 shows that the basic dual-branch network still exhibits clear misclassification in small regions and multi-class junctions, together with discontinuous boundaries. After DSDM is added, the contours of slender strands are preserved more completely. The DS-MobileViT Block produces more coherent predictions in larger regions, and the subsequent introduction of DVAS reduces local misclassification among adjacent angle classes. These visual changes are generally consistent with the quantitative results in Table 4.

#### 4.2.3. DVAS Weight and Angle-Class Confusion Analysis

To explain the selection of the directional auxiliary-loss weight λ_dir, values of 0, 0.05, 0.10, and 0.20 are compared while keeping the network structure, training parameters, and dataset split unchanged. The results are shown in Table 5.

As shown in Table 5, model performance does not vary monotonically with *λ*_dir_. When *λ*_dir_ = 0.05, the evaluation metrics do not improve consistently; when the weight is increased to 0.20, some metrics decline, indicating that an excessively strong directional constraint may interfere with the main segmentation task. In comparison, *λ*_dir_ = 0.10 yields the highest mIoU, Acc, and Dice values; therefore, *λ*_dir_ is set to 0.1 in this study.

**Figure 12:**
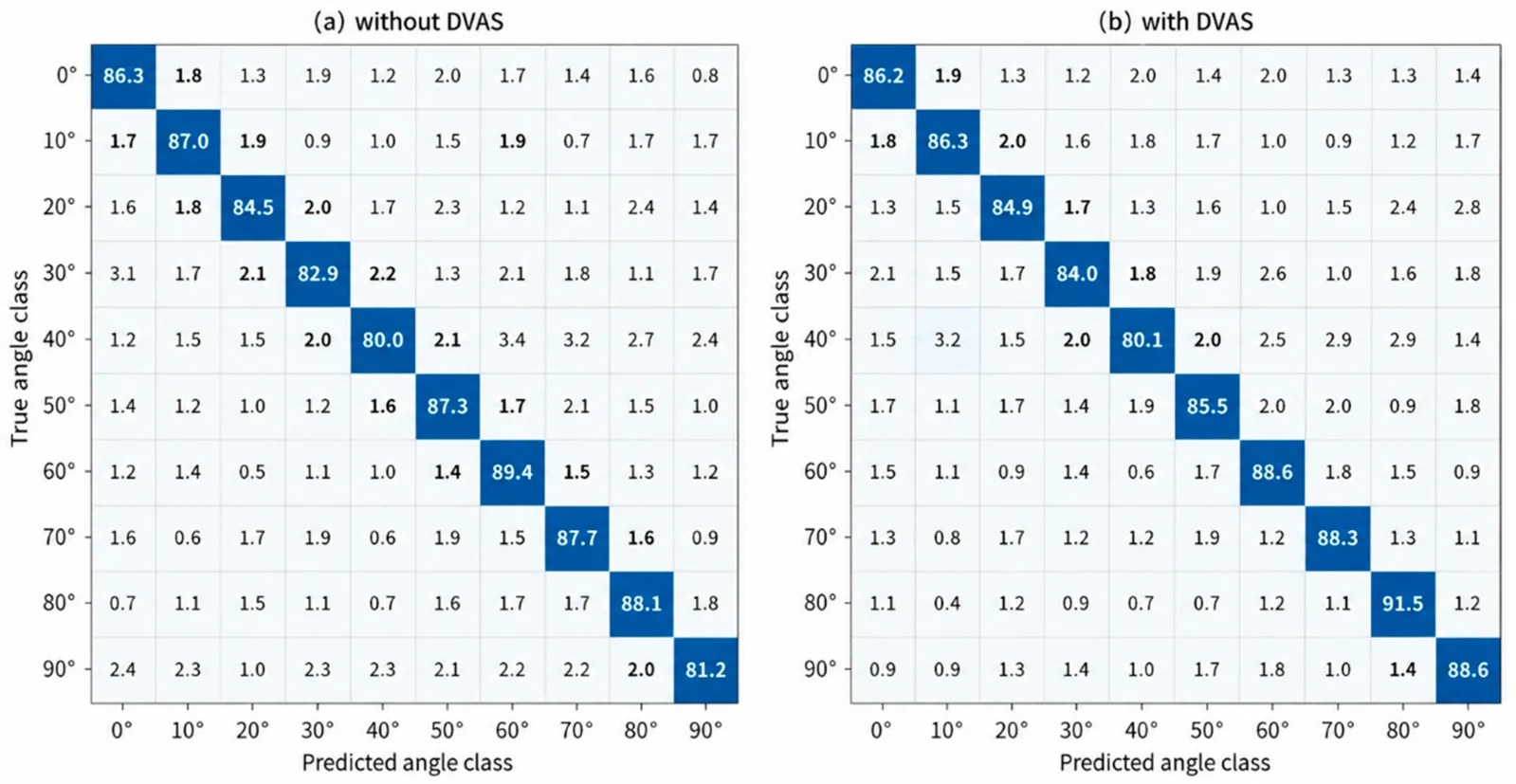
Angle-class confusion matrices before and after introducing DVAS. (**a**) Without DVAS; (**b**) with DVAS. Diagonal entries indicate correct classifications, while bold off-diagonal entries indicate misclassifications between adjacent angle classes.

As shown in Figure 12, after introducing DVAS, the mean value of the diagonal elements increases from 85.44% to 86.40%, whereas the mean confusion rate between adjacent angle classes decreases from 3.24% to 3.00%, corresponding to a relative reduction of approximately 7.4%. The recognition results for the 30°, 70°, 80°, and 90° classes improve more noticeably, although some fluctuations remain for several angle classes.

#### 4.2.4. Ablation Experiment on Feature-Fusion Methods

To independently analyze the contribution of BGFM, only the dual-branch feature-fusion method is replaced while the detail branch, semantic branch, DSDM, DS-MobileViT, DVAS, training settings, and dataset split remain unchanged. Four fusion methods are compared: element-wise addition, channel concatenation followed by a 1 × 1 convolution, channel concatenation followed by a 1 × 1 convolution and the Convolutional Block Attention Module (CBAM), and the proposed BGFM.

As shown in Table 6, BGFM achieves an mIoU of 0.8532, which is 1.75, 0.86, and 0.38 percentage points higher than element-wise addition, concatenation followed by a 1 × 1 convolution, and CBAM-based fusion, respectively. Acc and Dice also reach their highest values. Compared with fixed addition or concatenation, the gating structure can adjust the fusion weights according to the feature responses of the two branches, resulting in slightly better performance than the other fusion methods.

### 4.3. Comparison Experiments

To further verify the effectiveness of DiBiNet, representative semantic segmentation models, including DeepLabv3 [23], DeepLabv3+ [24], U-Net [25], U-Net++ [26], Swin Transformer [27], LSTFormer [28], and BiSeNetV2, are selected for comparison. All models are trained and tested using the same dataset split, and the results are shown in Table 7.

**Figure 13:**
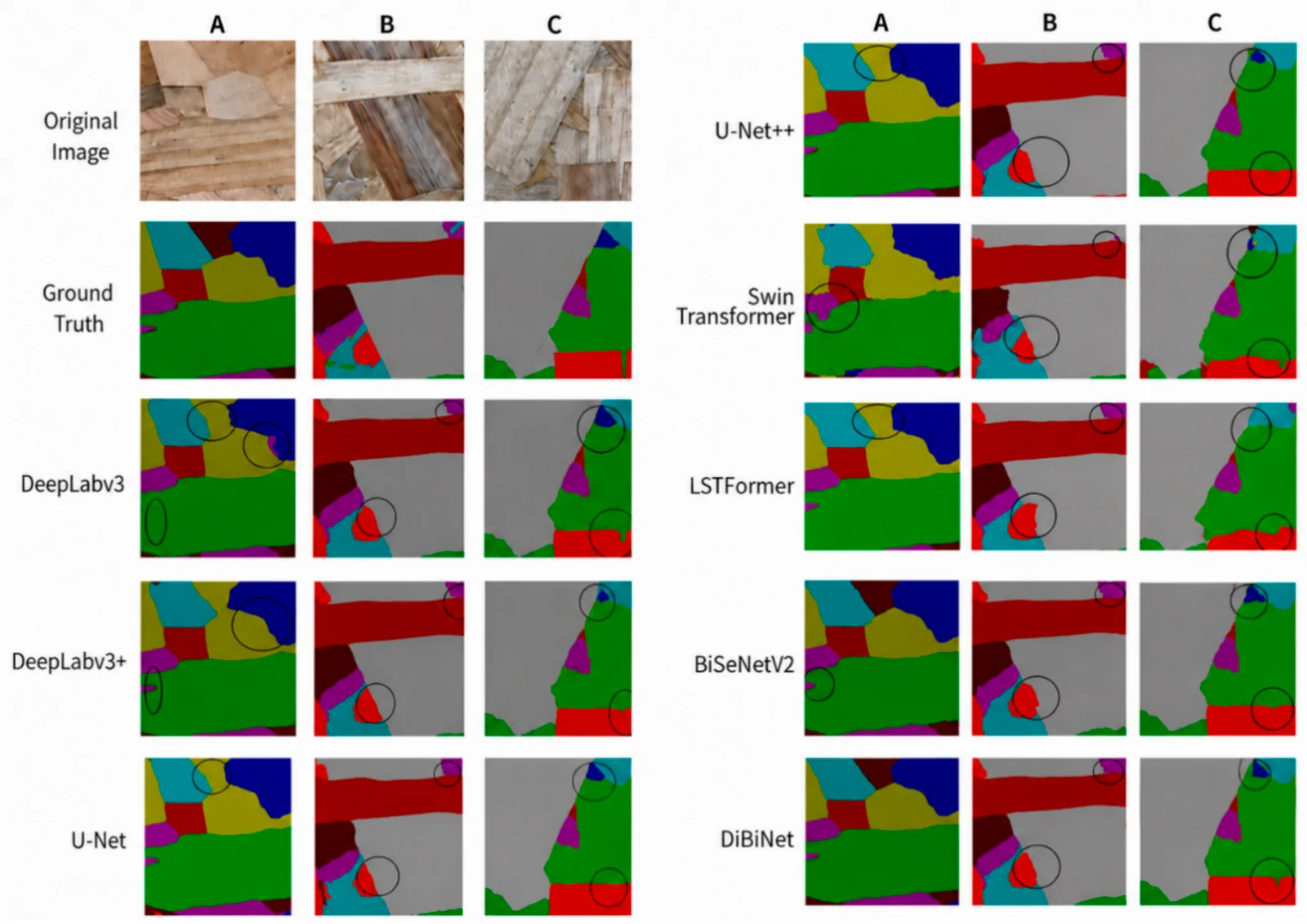
Comparison of segmentation results of different models. A–C denote three representative test samples; circles highlight representative error regions; different colors represent different angle classes and use the same color coding as the ground-truth maps.

As shown in Table 7, DiBiNet achieves mIoU, Acc, and Dice values of 0.8532, 0.8823, and 0.8623, respectively, and provides the best overall results among the compared models. Its mIoU is 1.31 percentage points higher than that of DeepLabv3+, its Acc is 2.01 percentage points higher than that of U-Net++, and its Dice is only 0.14 percentage points higher than that of DeepLabv3+. Therefore, the advantage of DiBiNet is mainly reflected in mIoU, Acc, and its overall performance across the three metrics, while the improvement in Dice is relatively limited. Figure 13 shows that DiBiNet preserves relatively complete boundaries in some small regions and multi-class junctions, although local misclassification remains. The standard deviations of all three DiBiNet metrics over three independent runs are below 0.001, indicating limited variation across runs.

To visually compare the performance of different models on the OSB surface-strand angle segmentation task, representative test samples are selected for qualitative analysis, as shown in Figure 13.

Figure 13 shows the segmentation results of different models on representative OSB surface images. All models can segment the main strand regions to some extent, but they differ in class misclassification, boundary discontinuity, and recognition stability for small regions. DeepLabv3 and DeepLabv3+ show partial boundary discontinuities when handling fine strands and blurred junctions. U-Net and U-Net++ preserve some local details but still show confusion in texture-similar regions. Swin Transformer and LSTFormer can model global information but are less stable in fine-boundary recovery. BiSeNetV2 has real-time advantages but may lose some detailed boundaries when dealing with complex overlapping strands.

In the circled regions, several comparison models show class omission, region expansion, or boundary shifts at multi-class junctions, small regions, and blurred boundaries. DiBiNet better preserves small-area angle classes and reduces misclassification and boundary discontinuity. This improvement mainly benefits from the combination of DSDM, the DS-MobileViT Block, BGFM, and DVAS. DSDM enhances directional boundary details, the DS-MobileViT Block supplements global context, BGFM adaptively fuses detail and semantic features, and DVAS introduces angular-continuity constraints during training.

These results indicate that DiBiNet improves segmentation stability in complex regions by jointly modeling detail information, semantic information, and angular-continuity constraints, while also providing more reliable segmentation results for subsequent angle-area proportion statistics.

Figure 13 further shows that the prediction of DiBiNet is largely consistent with the ground truth for Sample A, whereas local errors are mainly concentrated in Samples B and C. In Sample B, the small purple region in the upper-right corner is slightly over-expanded, and the boundaries of the red and cyan regions in the lower-left area differ from the ground truth. In Sample C, local misclassification occurs at the junction of the blue, cyan, and green regions in the upper part, and a boundary shift is also observed between the red and green regions in the lower-right area. These results indicate that DiBiNet may still produce local misclassification or boundary deviation for small-area classes and multi-class boundary junctions, possibly because such regions contain relatively few pixels and have complex boundary shapes. In addition, when the true strand orientation lies close to the boundary between two adjacent 10° classes, the discrete labels themselves are somewhat ambiguous, which may also cause confusion between neighboring angle classes. Overall, the model can accurately segment the main regions, but limitations remain in handling small local regions, complex junctions, and samples near angle-class boundaries.

The final objective of this study is to calculate the area proportions of strands at different angles. Based on the model segmentation results, the number of pixels in each angle class is counted, and its proportion among all strand pixels is calculated to obtain the angle distribution of the OSB surface-strands:

(15) $$P_{c}=\frac{N_{c}}{\sum_{k=1}^{10}N_{k}},\;c=1,2,\ldots ,10$$

where *N_c_* denotes the number of predicted pixels in the c-th angle class, and the denominator is the sum of predicted pixels over all foreground angle classes. The background class is excluded from the angle-proportion statistics.

**Figure 14:**
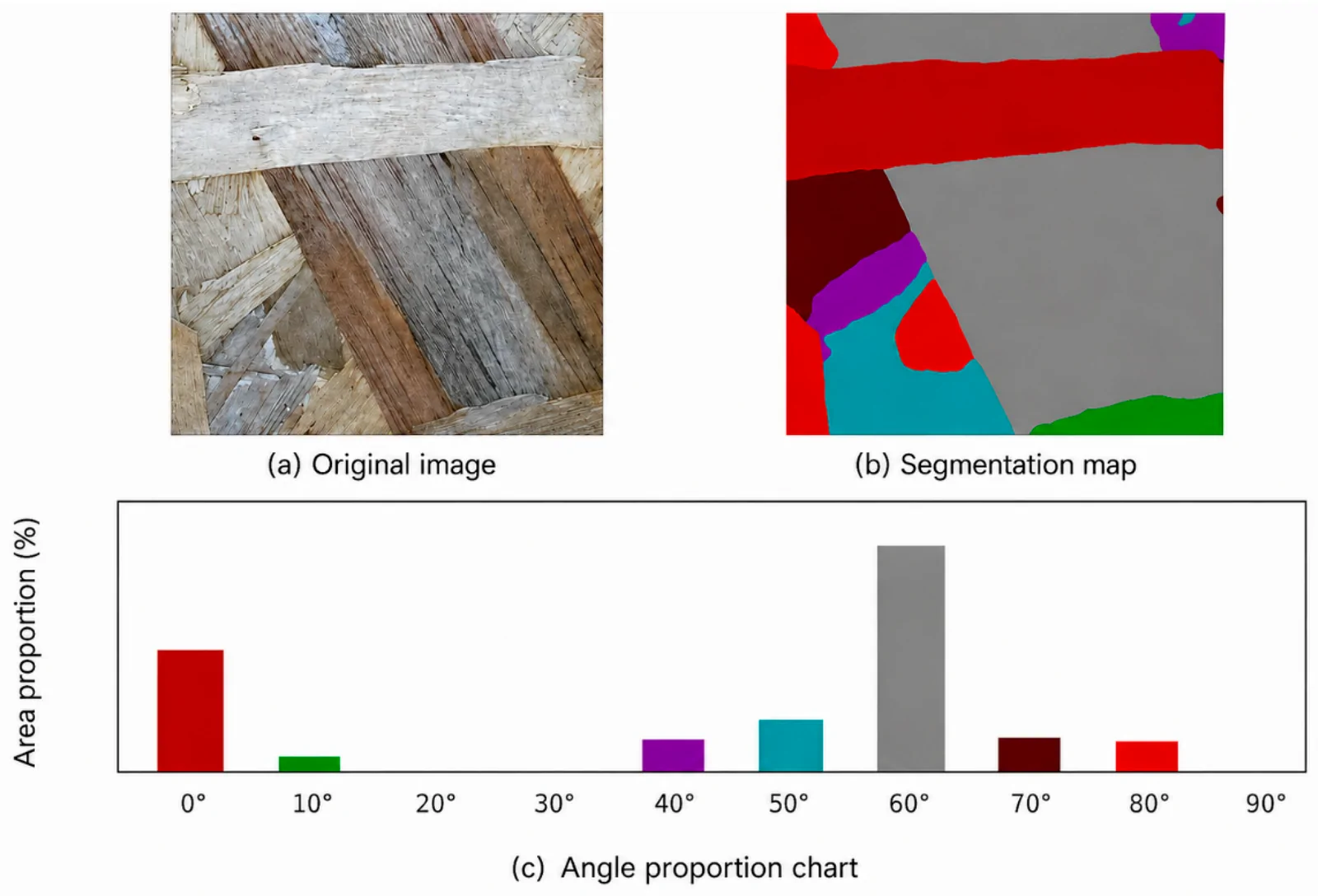
Segmentation result and angle-class proportion statistics of OSB surface-strands. (**a**) Original image; (**b**) segmentation map; (**c**) angle-proportion chart. The same color coding is used for the corresponding angle classes in (**b**) and (**c**).

Based on the above statistical analysis, the area proportions of different strand angle classes in this example are shown in Figure 14.

### 4.4. Edge Deployment Verification

To verify the practical applicability of the proposed model, DiBiNet is further deployed using the Rockchip Neural Network (RKNN) software toolchain [29] on the RK3588 edge-computing platform [30]. During deployment, the trained PyTorch 2.0.1 model is first exported to Open Neural Network Exchange (ONNX) format, then converted into an RKNN model using RKNN-Toolkit2 v2.3.2, and finally executed on the board with RKNN-Toolkit-Lite2 v2.3.2 on the neural processing unit (NPU). To meet the real-time requirements of edge inspection, 8-bit integer (INT8) and INT8 + 16-bit floating-point (FP16) mixed-quantization strategies are tested. DiBiNet contains 10.19 M parameters and requires 18.13 giga floating-point operations (GFLOPs). The model architecture remains unchanged under different precision modes, and the corresponding deployment performance is reported in Table 8.

During deployment testing, the input image size is fixed at 512 × 512. The reported inference time is the average time required for single-frame forward inference and excludes image loading, result visualization, and file saving. Compared with the original PyTorch model, the non-quantized RKNN model shows a slight mIoU decrease from 85.32% to 85.06%, which may be related to numerical differences introduced during ONNX export and RKNN model conversion. As shown in Table 8, INT8 quantization significantly shortens inference time and increases FPS, but causes a noticeable decrease in segmentation accuracy. The INT8 + FP16 mixed-quantization strategy maintains an mIoU of 83.87% while achieving a better balance between segmentation accuracy and inference speed. These results indicate the practical deployment potential of DiBiNet for OSB surface inspection on edge devices.

## 5. Conclusions

To address complex strand textures, blurred boundaries, local adhesion between adjacent strands, and subtle differences among adjacent angle classes in OSB surface images, this study proposes DiBiNet, a direction-aware dual-branch semantic segmentation network. Designed for the characteristics of OSB surface-strand angle segmentation, DiBiNet uses a high-resolution detail branch to preserve boundary and texture information and a low-resolution semantic branch to extract high-level semantic features. The DSDM enhances directional boundary details, the DS-MobileViT Block supplements global contextual information, and BGFM adaptively fuses detail and semantic features. In addition, DVAS maps discrete angle labels into continuous direction-vector representations, allowing the model to learn the continuity among angle classes during training.

Experimental results show that DiBiNet achieves an mIoU of 0.8532, an Acc of 0.8823, and a Dice coefficient of 0.8623 on the self-constructed OSB surface-strand angle segmentation dataset, outperforming several representative semantic-segmentation models overall. Ablation results demonstrate that DSDM, the DS-MobileViT Block, and DVAS each contribute to performance improvement and have complementary effects. After INT8 + FP16 mixed quantization, the model achieves an NPU inference speed of 34.0 frames per second (FPS) on the RK3588 platform, indicating that the proposed method has practical application potential in vision-based sensing and edge AI inspection.

Future work can be carried out from two aspects: data expansion and analysis methods. First, OSB surface images collected from different tree species, manufacturing processes, and acquisition environments can be expanded to improve model generalization. Second, connected-component analysis and geometric feature extraction can be combined with the segmentation results to further evaluate strand morphology, spatial distribution, and orientation consistency, thereby providing more direct quantitative support for OSB mat formation quality assessment and process optimization.

## Figures and Tables

**Table 1:** Distribution of annotated strand instances across angle classes.

Angle Class	Class Index	Annotated Instances	Proportion/%
0°	1	10,480	18.00
10°	2	11,230	19.29
20°	3	9510	16.33
30°	4	7120	12.23
40°	5	4920	8.45
50°	6	3880	6.66
60°	7	3450	5.93
70°	8	2900	4.98
80°	9	2450	4.21
90°	10	2280	3.92
Total	—	58,220	100.00

**Table 2:** Experimental environment configuration.

Hardware environment	CPU	Intel Core i7-11700KF
	GPU	NVIDIA RTX4090
	RAM	24 GB
Software environment	OS	Win11
	CUDA	11.8
	cuDNN	8.7.0
	Python	3.8.0
	torch	2.0.1
	torchvision	0.15.2

**Table 3:** Ablation results of data augmentation strategies.

Augmentation Method	mIoU	Acc	Dice
Conventional augmentation	0.8465	0.8768	0.8584
Conventional Copy–Paste	0.8482	0.8791	0.8601
Angle-semantic-consistent Copy–Paste	0.8532	0.8823	0.8623

**Table 4:** Ablation results of model modules.

Config	Basic Dual-Branch Network	DSDM	DS-MobileViT	DVAS	mIoU	Acc	Dice
(1)	✓				0.8213	0.8541	0.8452
(2)	✓	✓			0.8312	0.8622	0.8502
(3)	✓		✓		0.8294	0.8607	0.8496
(4)	✓	✓	✓		0.8483	0.8773	0.8601
(5)	✓	✓	✓	✓	0.8532	0.8823	0.8623

**Table 5:** Sensitivity analysis of the direction-loss weight λ_dir.

λ_dir_	mIoU	Acc	Dice
0	0.8483	0.8773	0.8601
0.05	0.8476	0.8781	0.8594
0.10	0.8532	0.8823	0.8623
0.20	0.8495	0.8768	0.8607

**Table 6:** Ablation results of different feature-fusion methods.

Fusion Method	mIoU	Acc	Dice
Element-wise addition	0.8357	0.8664	0.8498
Concatenation + 1 × 1 convolution	0.8446	0.8759	0.8571
Concatenation + 1 × 1 convolution + CBAM	0.8494	0.8792	0.8605
BGFM	0.8532	0.8823	0.8623

**Table 7:** Comparison results of different models.

Model	mIoU	Acc	Dice
DeepLabv3	0.8313 ± 0.0013	0.8502 ± 0.0011	0.8544 ± 0.0012
DeepLabv3+	0.8401 ± 0.0011	0.8550 ± 0.0009	0.8609 ± 0.0009
U-Net	0.8210 ± 0.0015	0.8552 ± 0.0013	0.8432 ± 0.0014
U-Net++	0.8385 ± 0.0010	0.8622 ± 0.0011	0.8523 ± 0.0010
Swin Transformer	0.8199 ± 0.0018	0.8524 ± 0.0016	0.8512 ± 0.0015
LSTFormer	0.8285 ± 0.0015	0.8621 ± 0.0014	0.8420 ± 0.0016
BiSeNetV2	0.8262 ± 0.0012	0.8540 ± 0.0011	0.8443 ± 0.0013
DiBiNet	0.8532 ± 0.0009	0.8823 ± 0.0008	0.8623 ± 0.0008

**Table 8:** Deployment performance test results on RK3588.

Precision Mode	Model Filesize/MB	Peakruntime Memory/MB	mIoU/%	Preprocessing Time/ms	NPUinference Time/ms	Postprocessing Time/ms	End-to-End FPS
No quantization	21.70	168.4	85.06	3.4	45.8	4.1	18.8
INT8	13.12	121.6	82.13	3.1	25.9	3.6	30.7
INT8 + FP16	13.93	136.2	83.87	3.2	29.4	2.8	28.2

## Data Availability

The data presented in this study are available from the corresponding author upon reasonable request. The data are not publicly available due to the large data volume and ongoing related research.
